# Person–Job Fit and Communication with Angry Patients Among Frontline Administrative Healthcare Staff: The Moderating Role of Job Tenure

**DOI:** 10.3390/healthcare14070919

**Published:** 2026-04-01

**Authors:** Simge Samanci

**Affiliations:** Medical Documentation and Secretarial Studies Program, Vocational School of Health Services, Hacettepe University, Ankara 06100, Turkey; simge_atakli@hacettepe.edu.tr

**Keywords:** person–job fit, communication skills, angry patients, frontline administrative healthcare staff, job tenure

## Abstract

**Highlights:**

**What are the main findings?**

- Person–job fit is positively associated with frontline administrative healthcare staff’s communication competencies with angry patients, across all subdimensions.
- Job tenure moderates the association between person–job fit and communication skills, while the association weakens at higher levels of tenure and becomes non-significant for creating effective messages.
- The association between person–job fit and communication competencies tends to be stronger among employees with shorter job tenure.

**What are the implications of the main findings?**

- Enhancing person–job fit may contribute to improving communication processes and reducing potential conflicts in healthcare settings.
- Considering job tenure may be important when developing strategies to improve communication competencies in emotionally demanding interactions.

**Abstract:**

**Background**: Effective communication with angry patients and their relatives is a critical determinant of patient satisfaction and service quality in healthcare settings. Medical secretaries, as frontline administrative healthcare staff, frequently manage emotionally demanding interactions with patients. However, limited research has examined the factors influencing their communication effectiveness in such situations. **Objectives**: This study investigates the association between person–job fit and frontline administrative healthcare staff’s communication competencies with angry patients and their relatives and examines the moderating role of job tenure in this relationship. **Methods**: A quantitative cross-sectional survey was conducted with 363 frontline administrative healthcare staff working in public and private healthcare institutions in Turkey, all of whom were medical secretaries. Pearson correlation analysis, simple linear regression, and Hayes’ PROCESS macro were employed to test the research hypotheses and examine the moderating role of job tenure. **Results**: The results indicated that person–job fit was significantly associated with all dimensions of communication competencies with angry patients, including displaying a positive approach (β = 0.464, *p* < 0.001), creating effective messages (β = 0.378, *p* < 0.001), anger anticipation and awareness (β = 0.366, *p* < 0.001), and anger management (β = 0.436, *p* < 0.001). In addition, job tenure moderated these relationships, with associations weakening at higher levels of tenure. Notably, for creating effective messages, the association became non-significant at higher tenure. **Conclusions**: Enhancing person–job fit—particularly among frontline administrative healthcare staff with shorter job tenure—may contribute to more effective communication, reduce potential conflicts, and better healthcare service quality. The study provides meaningful theoretical and managerial implications for healthcare organizations seeking to foster safer and more effective working environments.

## 1. Introduction

Violent incidents in healthcare institutions remain difficult to fully document, and their true extent is often unknown because many incidents go unreported. Nevertheless, the observed significant increase in violence is considered a critical problem that negatively affects healthcare workers’ working conditions, reduces occupational motivation, and undermines the trust relationship between patients and healthcare professionals [1,2]. In particular, interactions with patients and their relatives who express anger represent one of the most challenging aspects of healthcare communication.

Anger, an emotion inherent in human nature, can negatively affect interpersonal communication, leading to conflicts [3] and, at more advanced levels, to aggressive behaviors [4,5] and workplace violence [6]. Therefore, in managing conflicts that may arise between healthcare professionals and patients or relatives who express anger within healthcare settings, effective communication is considered to play a crucial role.

Healthcare secretaries act as a liaison between patients and physicians and represent frontline administrative staff within healthcare organizations. Given this role, they are frequently exposed to patients and their relatives who may express anger for various reasons throughout administrative healthcare processes. Consequently, by responding to these expressions of anger, medical secretaries often assume a buffering role between patients and healthcare professionals. This position places frontline administrative healthcare staff in a particularly high-risk role in terms of workplace violence. The literature indicates negative relationships between healthcare workers’ communication skills and patient–healthcare worker conflicts; in other words, as communication skills increase, levels of conflict and the likelihood of workplace violence decrease [1,7]. The underlying reason for this relationship is that accurate and effective communication can have a calming effect, even among patients with psychological problems [1]. Therefore, possessing high communication skills during interactions with patients and relatives expressing anger is important for frontline administrative healthcare staff in managing tension and maintaining conflict at manageable levels.

Moreover, healthcare workers’ ability to communicate effectively with patients and relatives who express anger is not limited to the technical training they receive [8]; individual and professional characteristics are also likely to significantly shape these skills. Previous studies have demonstrated that communication skills training increases healthcare workers’ self-efficacy in coping with aggressive behaviors [9]. In addition, while previous research has examined communication with difficult or aggressive patients in healthcare settings [8,10], limited attention has been given to frontline administrative healthcare staff, who frequently serve as the first point of contact in such interactions. However, for communication skills to be applied sustainably and effectively, employees’ personal resources must also be strong. In this context, person–job fit (PJF) refers to the alignment between individuals’ personal characteristics and job requirements and is considered an important personal resource that helps employees cope with job-related challenges [11]. In addition, job tenure may function as a professional resource that may contribute to employees’ ability to accurately assess situations, regulate emotional responses, and develop effective communication strategies [12]. Accordingly, both PJF and professional experience may be associated with the effectiveness of communication with angry patients. In this context, the study examines the communication abilities of frontline administrative healthcare staff who interact with angry patients. At the same time, the study examines the relationship between the skills used in communication with angry patients and PJF. Furthermore, based on the Conservation of Resources (COR) theory, the study considers the possible moderating role of job tenure in these relationships. From a COR perspective, communication competencies with angry patients can be viewed as an important capacity that helps prevent resource loss and protect existing resources [13]. Accordingly, PJF is conceptualized in this study as a perceptual and functional personal resource that facilitates individuals’ management of job demands within the framework of COR theory. In this regard, by focusing on frontline administrative healthcare staff, the present study responds to the research call articulated in the literature. Despite their critical role in healthcare service provision, significant gaps remain in the literature concerning frontline administrative healthcare staff.

Prior research consistently links PJF to favorable work attitudes and performance outcomes, such as job satisfaction and performance [14,15,16]. However, most existing studies have focused on different occupational groups. Consequently, the relationship between PJF and frontline administrative healthcare staff’s ability to manage interactions with angry patients and their relatives, as well as the moderating role of job tenure in this association, has received limited attention in the literature. Furthermore, the inclusion of job tenure in the research model addresses previous criticisms in the literature regarding the omission of this variable in many empirical studies [17] and contributes by examining the moderating role of job tenure in communication competencies. Understanding how job tenure shapes employee behavior is critical for explaining workplace interactions [18]. Therefore, the findings of this study are expected to provide valuable insights into the individual and professional resources that strengthen frontline administrative healthcare staff’s communication competencies in interactions with angry patients. Within this framework, the present study aims to examine the following questions:

1. How is PJF associated with frontline administrative healthcare staff’s communication competencies with angry patients?
2. How does job tenure moderate the relationship between PJF and communication competencies with angry patients?

## 2. Conceptual Model and Hypotheses

### 2.1. The Concept of Person–Job Fit

PJF, which is based on person–environment fit theory, emphasizes the alignment between individuals and their work environments and holds an important place in organizational psychology [19]. Within this theoretical perspective, person–job fit refers to the congruence between job demands and individuals’ skills, knowledge, and abilities [20]. According to fit theory, the greater alignment between individual characteristics and job requirements enhances the effectiveness of the individual–job relationship [21]. Within this context, the knowledge, skills, and abilities possessed by individuals constitute the core components of person–job fit and are directly associated with task performance [22,23,24]. Previous empirical studies have shown that person–job fit is associated with employee performance [19,24].

Individuals who work in environments aligned with their knowledge, skills and needs tend to exhibit more positive behaviors, thereby promoting the emergence of positive outcomes at both the individual and organizational levels [19,25]. In the literature, person–environment fit is examined across three main dimensions: person–organization fit, person–group fit, and PJF [26]. Among these dimensions, empirical evidence suggests that PJF has a stronger effect on task performance compared to other types of fit [27]. Accordingly, in the present study, frontline administrative healthcare staff’s communication skills with patients and relatives expressing anger are examined within the framework of PJF.

PJF plays a significant role in employees’ work-related experiences and reflects the alignment between job demands and employees’ knowledge, skills, and personal characteristics [14,28,29]. Given the significance attributed to the relationships between PJF and organizational variables, this construct has been examined in numerous studies in relation to various organizational outcomes. Prior research consistently links PJF to favorable work attitudes and performance outcomes [15,16,30,31,32,33,34].

From a healthcare communication perspective, employees in the service sector are prone to engaging in emotional labor [35], and healthcare workers are required to use emotional labor strategies when interacting with patients and their relatives [36]. Managing workplace conflict, manipulation, and aggression requires the regulation and control of emotions through emotional labor strategies [37]. On the other hand, organizations in the service sector typically expect employees to display organizationally desired emotions during interactions with customers [35]. When the emotions that employees are required to display during communication do not reflect their genuine feelings, this may lead to work alienation [38], as well as increased burnout and turnover intentions [39].

### 2.2. Communication Competencies with Angry Patients

Anger constitutes the underlying emotional basis of angry behaviors [40]. The literature emphasizes that effective communication skills are among the key determinants in preventing conflicts between healthcare professionals and patients [10], and that the most effective strategy for coping with angry patients is establishing accurate and effective communication [41]. Moreover, it is well documented that ineffective communication between patients and healthcare professionals may increase patients’ levels of anger, escalate the interaction process, and ultimately lead to workplace violence at more advanced stages [10].

Kibaroğlu et al. (2024) conceptualized healthcare professionals’ competence in managing interactions with patients and their relatives expressing anger across four main dimensions: displaying a positive approach (DPA), creating effective messages (CEM), anger anticipation and awareness (AAA), and anger management (AM) [42]. Within this framework, the dimension of DPA is based on the ability to respond to patients’ or their relatives’ negative emotional reactions through an empathetic and constructive communication style. This is because low levels of PJF may lead professionals to become desensitized toward service recipients, resulting in service delivery—expected to be grounded in emotional labor—taking on a forced, superficial, and insincere character [43]. In contrast, healthcare professionals with high levels of PJF are expected to display positive emotions during service delivery to patients and their relatives, as their internal feelings are more congruent with the emotions they are expected to express in the work environment. Accordingly, the first hypothesis was developed:

**H1.**  *PJF is positively associated with frontline administrative healthcare staff’s ability to DPA*.

The second dimension, CEM, refers to healthcare professionals’ ability to recognize both verbal and nonverbal cues conveyed by patients and their relatives during communication, as well as to appropriately regulate their own communication elements in accordance with the situation. The literature highlights that effective communication is associated with patients’ perceptions and trust in healthcare services through healthcare professionals’ verbal and nonverbal behaviors [44]. It is also known that the flexible and context-appropriate use of verbal and nonverbal communication elements enhances communication effectiveness in healthcare service delivery [45]. Furthermore, nonverbal messages conveyed during communication processes serve a complementary function to verbal messages and can influence recipients’ evaluations [46]. Within this context, perceiving one’s role as congruent with personal skills and interests has been shown to support the creation of more effective messages in communication processes [47]. Accordingly, employees with high levels of PJF are expected to more accurately interpret others’ messages and to structure their own verbal and nonverbal messages in a manner appropriate to the situation. Based on this theoretical framework, PJF is expected to strengthen the ability to CEM in communication with patients and relatives expressing anger. Accordingly, the second hypothesis was formulated:

**H2.**  *PJF is positively associated with frontline administrative healthcare staff’s ability to CEM*.

The third dimension, AAA, refers to the ability to recognize increases in patients’ or their relatives’ levels of anger at an early stage and to take preventive measures accordingly. Retaliating against or directly responding to customers’ mistreatment is generally not considered appropriate for service employees [48]. In this respect, PJF may be considered to serve a protective function in service contexts characterized by intense emotional demands. From this perspective, the following hypothesis was developed:

**H3.**  *PJF is positively associated with frontline administrative healthcare staff’s AAA skills*.

The final dimension, AM, refers to healthcare professionals’ ability to regulate their emotions and behaviors in line with professional role requirements when interacting with patients and relatives who express anger, without internalizing the anger displayed during these encounters. External stimuli in hospital environments—such as noise, crowding, long waiting times, and uncertainty—combined with differences in patients’ individual perceptions and cognitive responses, may rapidly trigger anxiety, anger, and dissatisfaction even in minor interactions, thereby leading to violent and conflictual behaviors directed toward healthcare professionals [49]. Such challenging conditions render the need for professional emotion regulation more salient in employees’ working lives.

Moreover, the relatively limited control service employees have over situations that elicit emotional responses, due to the nature of their job roles, further underscores the critical importance of professional emotion regulation capacity [50]. Accordingly, employees with high levels of PJF are expected to perform this emotion regulation process with less strain due to the congruence between job requirements and individual characteristics, and to develop more effective and professional responses to anger-provoking interactions. Based on this rationale, a testable hypothesis was proposed:

**H4.**  *PJF is positively associated with frontline administrative healthcare staff’s AM skills*.

### 2.3. The Moderating Role of Job Tenure

In this study, job tenure is examined as a moderator of the relationship between PJF and interactions with patients expressing anger. COR theory posits that individuals’ resources may vary depending on contextual conditions and that, when resource loss occurs, individuals are motivated to acquire additional resources to compensate for such losses [13]. From this perspective, considering differences in employees’ levels of job tenure, it can be argued that employees possess varying amounts and types of resources, and that job tenure may be regarded as an important individual factor determining the level of resources accessible to individuals. Based on this argument, employees with longer job tenure may possess greater resources as a result of accumulated professional knowledge and experience over time; therefore, the association between PJF and communication skills may be relatively weaker within this group. Accordingly, it is assumed that the positive association between PJF and all subdimensions of communication competencies in anger-related patient and family interactions will be more pronounced among employees with lower levels of job tenure.

**Figure 1:**
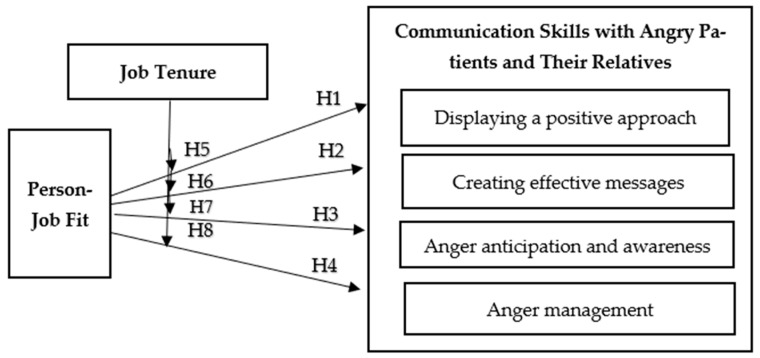
Research Model.

In this context, the present study proposes a conceptual research model examining the moderating role of job tenure in the relationship between frontline administrative healthcare staff’s PJF and their communication skills with patients and relatives expressing anger (Figure 1). Based on this conceptual model, the following hypotheses were developed:

**H5.**  *Job tenure moderates the association between PJF and DPA*.

**H6.**  *Job tenure moderates the association between PJF and CEM*.

**H7.**  *Job tenure moderates the association between PJF and AAA*.

**H8.**  *Job tenure moderates the association between PJF and AM*.

## 3. Research Design

### 3.1. Characteristics and Procedure

The present research is a cross-sectional field study. The population of the research consists of healthcare personnel working as frontline administrative healthcare staff in healthcare institutions in Turkey. Since the exact number of frontline administrative healthcare staff in Turkey is not known, sample size adequacy was evaluated using G*Power 3.1 software. Assuming a medium effect size (f^2^ = 0.15), an alpha level of 0.05, and a statistical power of 0.80, the analysis indicated that the final sample of 363 participants was sufficient for the proposed analyses. Accordingly, convenience sampling and snowball sampling methods were employed to reach appropriate participants in a timely and cost-effective manner [51,52]. Written informed consent was obtained electronically from all participants prior to their participation in the study by requiring them to indicate their consent before accessing the online questionnaire. Participation was voluntary, and respondents were informed about confidentiality and anonymity, and no financial or material incentives were provided. The online questionnaire, prepared via Google Forms, was distributed via LinkedIn and WhatsApp. Sharing online survey links across different social networking platforms has been widely recognized as an effective method by many researchers [51,53]. Inclusion criteria required participants to be currently employed as frontline administrative healthcare staff in public or private healthcare institutions and to have at least one year of work experience. Responses that were incomplete or did not meet these criteria were not included in the study. A total of 363 frontline administrative healthcare staff participated in the study.

### 3.2. Ethical Considerations

The necessary ethical approval to conduct the study was obtained from the Social and Human Sciences Ethics Committee of Hacettepe University (Approval No: 08, Date: 22 April 2025). The research was carried out in compliance with the ethical principles outlined in the Declaration of Helsinki. In addition, the participating frontline administrative healthcare staff were informed that all their ethical rights would be protected, and they were included in the study after their informed consent had been obtained.

### 3.3. Data Analysis

Data were analyzed using IBM SPSS Statistics (version 24.0, IBM Corp., Armonk, NY, USA), IBM SPSS AMOS (version 26.0, IBM Corp., Armonk, NY, USA), and the PROCESS macro for SPSS (version 4.3, Andrew F. Hayes). As the assumption of normality was satisfied, parametric tests were employed in the analyses. For continuous variables, mean, standard deviation, minimum, and maximum values were used, whereas frequency and percentage values were reported for categorical variables. Internal consistency was evaluated using Cronbach’s alpha. Relationships among the variables were examined using Pearson correlation analysis. In addition, simple linear regression analysis was used to test the associations between the variables and the subdimensions as well as the total score of communication skills with angry patients and their relatives. The moderating effect of job tenure on the relationship between PJF and communication skills with patients and relatives expressing anger was examined using Model 1 of the PROCESS macro developed by Hayes (2017) [54]. Separate models were developed and analyses were conducted for the DPA, CEM, AAA, and AM variables. For each model, PJF was included as the independent variable (X), while job tenure was included as the moderator (W). Additionally, the interaction term (PJF × job tenure) was also included in the model to examine the moderating role of job tenure. Job tenure was categorized into three ordered groups (1–5 years, 6–10 years, 11 years and above) and treated as an ordinal moderator reflecting increasing levels of professional experience in the PROCESS moderation model. In addition, as the data were collected via self-report, common method variance (CMV) was evaluated using Harman’s single-factor test. The results indicated that the first factor accounted for 43.58% of the total variance, which is below the commonly accepted threshold of 50% [55]. Therefore, common method bias is unlikely to pose a serious concern in this study. In addition, several procedural remedies were applied to reduce common method bias, including ensuring anonymity and confidentiality of responses.

### 3.4. The Data Collection Tools

The measurement instruments used in this study are described below.

Person–Job Fit Scale: In the original validation study, the scale demonstrated acceptable model fit (χ^2^/df = 1.714, CFI = 0.85, TLI = 0.85, RMSEA = 0.078) [56]. Its validity and reliability have also been supported by subsequent studies [57]. The scale is a single-dimensional instrument consisting of four items and is measured on a five-point Likert scale (1 = Not at all appropriate, 5 = Completely appropriate). Higher scores obtained from the scale indicate higher levels of PJF among participants. PJF was measured as perceived (subjective) fit, rather than objective fit. The scale includes items such as “To what extent are your knowledge, skills, and abilities appropriate for the conditions of the job you perform?” and “To what extent does the job you perform satisfy your needs?”. In the original study, the Cronbach’s alpha coefficient was presented as 0.87, indicating an acceptable level of internal consistency.

Communication Skills with Angry Patients and Their Relatives Scale: The scale was developed by Kibaroğlu et al. (2024) [42]. The scale consists of 19 items measured on a five-point Likert scale (1 = Strongly disagree, 5 = Strongly agree). The validity and reliability of the scale were established by the original authors (χ^2^/df = 3.812; CFI = 0.956; GFI = 0.987; RMSEA = 0.041; TLI = 0.966). In addition, the scale comprises four dimensions (DPA, CEM, AAA, AM). Higher scores on the scale indicate higher levels of communication skills with patients and relatives expressing anger. Sample items include statements such as “I maintain my patience toward angry patients/their relatives” and “When I encounter an angry patient or relative, I approach them in a calm and understanding manner.” In the original study, the overall Cronbach’s alpha coefficient was reported as 0.89, and the reliability coefficients for the subdimensions were shown to be above 0.75. These findings indicate strong internal consistency.

Job Tenure: In this study, job tenure was operationalized as total professional experience measured in years, rather than tenure within a single organization [58]. This measurement approach has been widely adopted in previous studies in the literature [25].

## 4. Results

### 4.1. Characteristics of the Participants

Table 1 presents the descriptive statistics of continuous variables, while Table 2 summarizes the categorical characteristics of the participants. The majority of the frontline administrative healthcare staff participating in the study were female (54.8%) and held a degree from a Vocational School of Health (86.5%). In addition, nearly half of the participants reported having 1–5 years of work experience (51.2%). On the other hand, most frontline administrative healthcare staff were single (79.1%) and were currently employed in the private sector (66.1%). Furthermore, the participating frontline administrative healthcare staff generally reported working in the outpatient clinic department (52.1%).

### 4.2. Results on the Validity of the Scales

#### 4.2.1. Internal Consistency and Convergent Validity

Cronbach’s alpha (α) and composite reliability (CR) were used to assess internal consistency reliability [59], with a threshold value of 0.70 being considered acceptable [60]. In this context, the Cronbach’s alpha value of the PJF scale was calculated as 0.909. The Cronbach’s alpha internal consistency coefficients for the subdimensions of the Communication Skills with Angry Patients and Their Relatives scale were found to be 0.932 for the DPA subdimension, 0.896 for the CEM subdimension, 0.799 for the AAA subdimension, and 0.895 for the AM subdimension. As shown in Table 3, factor loadings (loadings ≥ 0.50), composite reliability coefficients (CR ≥ 0.70), and average variance extracted (AVE ≥ 0.50) values were above the recommended threshold levels [61]. These results indicate that convergent validity was established and that the scales demonstrated high internal consistency.

#### 4.2.2. Discriminant Validity

Discriminant validity was assessed using the Fornell–Larcker criterion [62] and the heterotrait–monotrait ratio (HTMT) [63]. According to the Fornell–Larcker criterion, the square root of the average variance extracted (AVE) for each construct should exceed its correlations with other constructs. The results indicated that this condition was not fully satisfied for some constructs. To provide a more robust assessment, HTMT values were also examined. According to Henseler et al. (2015), HTMT values should be below the threshold of 0.90 to establish discriminant validity [63]. Results indicate that some HTMT values exceed this threshold and that there is a high level of association between some dimensions. These results may suggest limited discriminant validity in psychometric terms. In practice, communication skills with angry patients and their relatives consist of behavioral and emotional processes that often emerge together and are closely interrelated. Therefore, this situation appears theoretically consistent. Accordingly, the dimensions can be considered as interrelated components of a broader communication competence structure. Prior studies have also emphasized that such behaviors tend to co-occur in practice [64]. Additionally, the use of a cross-sectional, single-source, and self-report design may have led to common method variance and similar response tendencies, thereby resulting in inflated associations among the variables [55,64]. Overall, the findings indicate that the scale has a coherent and theoretically meaningful structure. Therefore, the original factor structure of the scale was retained.

#### 4.2.3. Assessment of Construct Validity

Confirmatory factor analysis was employed to assess the construct validity of the measurement instruments. In evaluating the goodness-of-fit of the factor structures, acceptable fit indices were taken into consideration (Table 4). Kabataş Yıldız and Çal (2025) indicated that, in the literature, a χ^2^/df value below 5, an RMSEA value below 0.08, GFI and AGFI values above 0.80, and other fit indices above 0.90 are considered acceptable [65]. The results of the analyses showed that the goodness-of-fit indices of the PJF scale were within acceptable limits (χ^2^/df = 3.33, CFI = 0.99, GFI = 0.99, AGFI = 0.95, RMSEA = 0.08), and no modifications were performed. On the other hand, the goodness-of-fit indices of the Communication Skills with Angry Patients and Their Relatives scale were found to be outside acceptable limits (χ^2^/df = 4.3, CFI = 0.92, GFI = 0.84, AGFI = 0.79, RMSEA = 0.09). Based on modification indices, a limited number (n = 8) of error covariances were added between items within the same factor measuring closely related aspects of the construct. These residual covariances were theoretically justified, as the items share conceptual and wording similarities and capture closely related dimensions of communication behavior. These modifications did not alter the original factor structure and were consistent with the theoretical conceptualization of the scale. Following these modifications, the goodness-of-fit indices reached acceptable levels (χ^2^/df = 3.02, CFI = 0.94, GFI = 0.86, AGFI = 0.82, RMSEA = 0.07). Similar residual covariances between items within the same construct have also been reported in previous confirmatory factor analysis studies when theoretically justified [66].

Table 5 presents the descriptive statistics of the scales and their subdimensions used in the study, as well as the results indicating the normality of the data distribution. As shown in the table, frontline administrative healthcare staff’s PJF levels were found to be slightly above the average ($\bar{x}$ = 3.40; SD = 0.96). Similarly, frontline administrative healthcare staff’s communication skills with patients and relatives expressing anger were also found to be above the average level of competence ($\bar{x}$ = 3.87; SD = 0.77). In addition, although the subdimensions of the scale generally exhibited similar levels, frontline administrative healthcare staff were found to be more proficient in DPA ($\bar{x}$ = 3.89; SD = 0.87) and CEM ($\bar{x}$ = 3.88; SD = 0.80). On the other hand, their skills in AAA ($\bar{x}$ = 3.86; SD = 0.78) and AM ($\bar{x}$ = 3.86; SD = 0.86) were relatively lower compared to the other dimensions. Overall, while the subdimensions yielded similar results, it was determined that frontline administrative healthcare staff demonstrated greater proficiency in DPA and CEM, whereas they showed comparatively less pronounced competence in AAA and AM.

Furthermore, in order to determine the appropriate type of correlation analysis to be employed, the distributional properties of the data were evaluated by examining skewness and kurtosis values (Table 5). Since the skewness and kurtosis values were within the range of ±2, the data were considered to be normally distributed [67]. Accordingly, the assumption of normality was satisfied.

### 4.3. Correlations of the Variables

Pearson correlation analysis was conducted to examine the relationships between PJF and the subdimension scores of the Communication Skills with Angry Patients and Their Relatives scale among frontline administrative healthcare staff. When evaluating the correlation coefficient (r), relationships with absolute values below 0.30 were considered weak, those between 0.30 and 0.50 were considered moderate, and those of 0.50 and above were considered strong [68]. In this context, as shown in Table 6, the correlation coefficients between the scales ranged from 0.37 to 0.46, all exceeding 0.30, indicating satisfactory and statistically significant positive correlations between the variables (*p* < 0.01). In addition, the correlations among some subdimensions are relatively high, suggesting strong associations among these dimensions (Table 6).

### 4.4. Results of the Hypothesis Test

Simple linear regression analysis was conducted to examine the association between frontline administrative healthcare staff’s PJF and their ability to manage interactions with patients and relatives who express anger, and the results are presented in Table 7.

In Model 1, PJF was significantly associated with the ability to DPA (β = 0.464, t = 9.963, *p* < 0.001), and frontline administrative healthcare staff’s PJF explained 21.6% of the variance in DPA scores (R^2^ = 0.216, *p* < 0.001). The unstandardized coefficient further indicated a positive association between PJF and DPA (B = 0.423). Thus, H1 was supported. Similarly, in Model 2, PJF was significantly associated with frontline administrative healthcare staff’s ability to CEM (β = 0.378, t = 7.752, *p* < 0.001), and the model significantly explained 14.3% of the variance in CEM (R^2^ = 0.143, *p* < 0.001). The unstandardized coefficient further indicated a positive association between PJF and CEM (B = 0.314). Therefore, H2 was supported. Model 3 showed that PJF was significantly associated with frontline administrative healthcare staff’s AAA skills (β = 0.366, t = 7.464, *p* < 0.001), and this model significantly explained 13.4% of the variance in AAA scores (R^2^ = 0.134, *p* < 0.001). The unstandardized coefficient further indicated a positive association between PJF and AAA (B = 0.298). Accordingly, H3 was supported. Finally, Model 4 indicated that PJF was significantly associated with frontline administrative healthcare staff’s AM skills (β = 0.436, t = 9.211, *p* < 0.001), explaining 19.0% of the variance in AM scores (R^2^ = 0.190, *p* < 0.001). The unstandardized coefficient further indicated a positive association between PJF and AM (B = 0.392). Thus, H4 was supported.

### 4.5. Post-Hoc Analyses

The moderating role of job tenure in the relationships among the variables in the model was examined. First, analyses were conducted using the PROCESS Macro to test the moderating role of job tenure in the relationship between PJF and communication skills with patients and relatives expressing anger. The results of the bootstrap-based regression analyses and the findings regarding the moderating role of job tenure are presented in Table 8.

**Figure 2:**
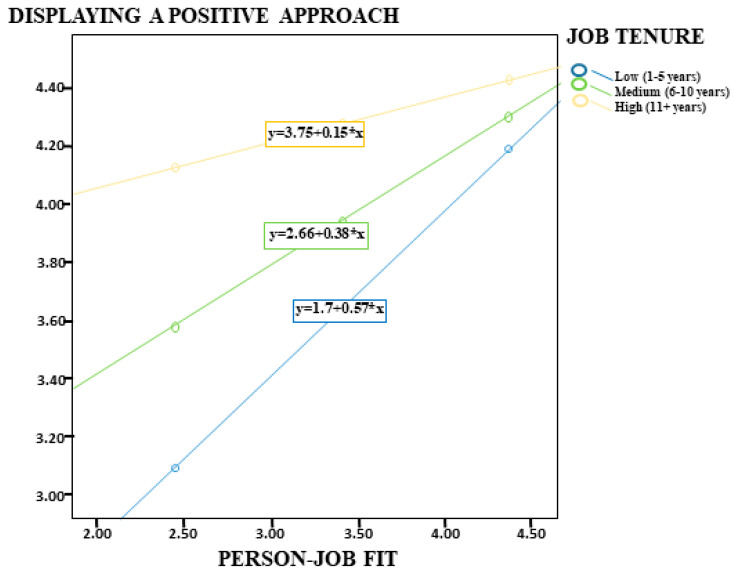
Job Tenure as a Moderator in the Relationship between Person–Job Fit and Displaying a Positive Approach.

According to the analysis results of Model 1 presented in Table 8, PJF was positively associated with employees’ levels of DPA (*p* < 0.001, [0.70, 1.10]). This finding indicates that higher levels of congruence between employees’ personal characteristics and job attributes are associated with more positive attitudes toward their work. On the other hand, the results showed that job tenure had a negative and statistically significant moderating role on this relationship (B = −0.33, *p* < 0.001); therefore, H5 was supported. This result suggests that as the level of job tenure increases, the positive association between PJF and DPA decreases (Figure 2). The findings further revealed that the association was stronger among employees with low job tenure (B = 0.57), weaker among those with moderate job tenure (B = 0.38), and weakest among employees with high job tenure (B = 0.15). The model findings indicated that the model explained 41% of the total variance (R^2^ = 0.41) and was statistically significant (F = 83.25, *p* < 0.001).

**Figure 3:**
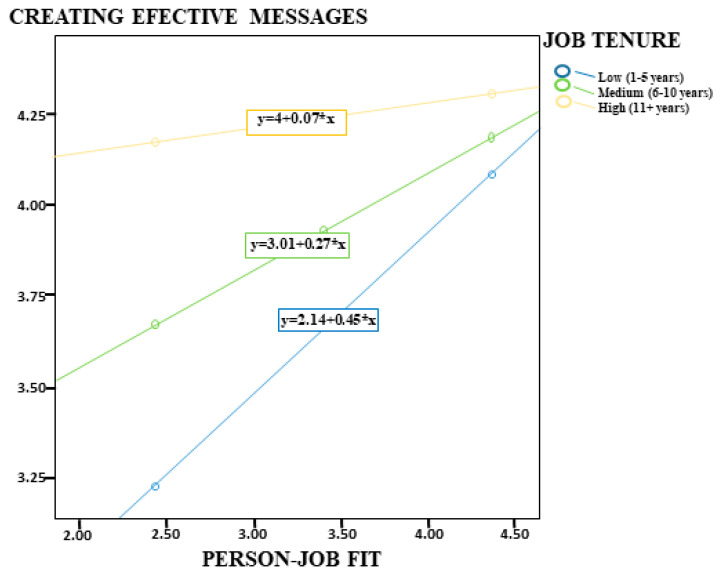
Job Tenure as a Moderator in the Relationship between Person–Job Fit and Creating Effective Messages.

In addition, the analysis results of Model 2 in the same table indicated that PJF was significantly associated with employees’ ability to CEM (*p* < 0.001, [0.55, 0.94]). This finding suggests that congruence between job requirements and individual attributes strengthens employees’ communication skills. Moreover, job tenure was found to have a negative and statistically significant moderating role on this relationship (B = −0.30, *p* < 0.001). This result indicates that as frontline administrative healthcare staff’s job tenure increases, the positive association between PJF and CEM decreases (Figure 3). Specifically, this association was stronger among employees with low job tenure (B = 0.45), weakened among those with moderate job tenure (B = 0.27), and was not statistically significant among employees with high job tenure (B = 0.07, *p* = 0.14). Therefore, H6 was supported for employees with low and moderate job tenure, but not supported for those with high job tenure. The model findings indicated that the model explained 34% of the variance (R^2^ = 0.34) and was statistically significant overall (F = 61.41, *p* < 0.001).

**Figure 4:**
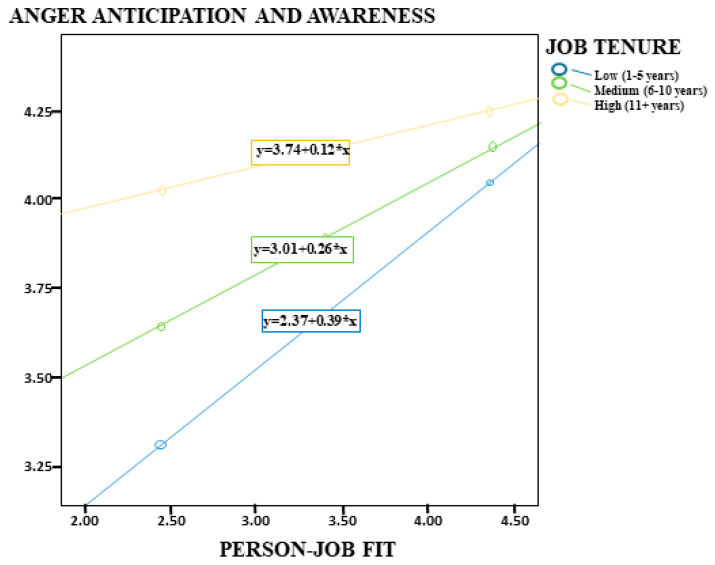
Job Tenure as a Moderator in the Relationship between Person–Job Fit and Anger Anticipation and Awareness.

The analysis results of Model 3 in the same table showed that PJF was significantly associated with employees’ levels of AAA (*p* < 0.001, [0.40, 0.80]). This finding indicates that PJF strengthens individuals’ emotional awareness. In addition, job tenure had a negative and statistically significant moderating role in this relationship (B = −0.21, *p* < 0.001); therefore, H7 was supported. This result suggests that as the level of job tenure increases, the positive association between PJF and AAA weakens. In other words, the association was stronger among individuals with low job tenure (B = 0.39), weaker among those with moderate job tenure (B = 0.26), and became even weaker among individuals with high job tenure (B = 0.12, *p* = 0.02) (Figure 4). The model findings indicated that the model explained 25% of the variance (R^2^ = 0.25) and was statistically significant (F = 40.84, *p* < 0.001).

**Figure 5:**
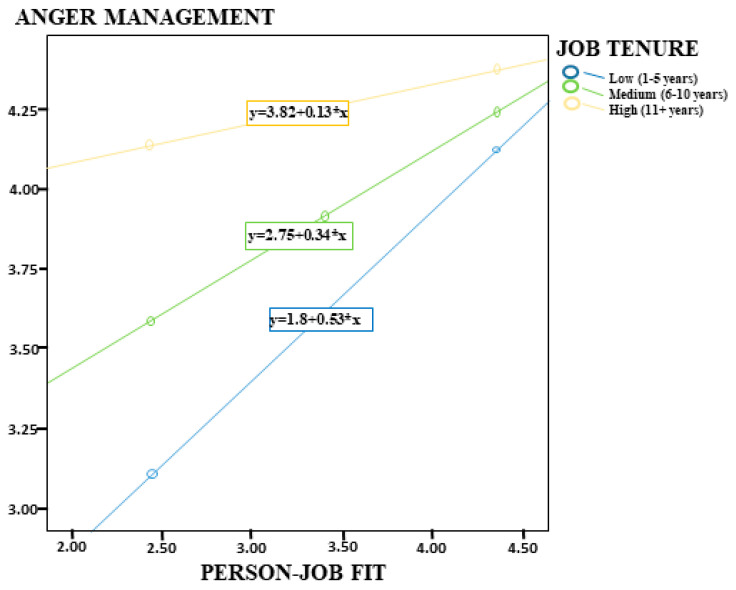
Job Tenure as a Moderator in the Relationship between Person–Job Fit and Anger Management.

Finally, the analysis results of Model 4 in the same table indicated that PJF was significantly associated with employees’ AM skills (*p* < 0.001, [0.65, 1.05]). On the other hand, job tenure was also found to have a negative and statistically significant moderating role on this relationship (B = −0.32, *p* < 0.001); thus, H8 was supported. This result indicates that as individuals’ job tenure increases, the positive association between PJF and AM skills decreases (Figure 5). Additional analyses showed that this association was stronger among employees with low job tenure (B = 0.53), weaker among those with moderate job tenure (B = 0.34), and relatively weak among employees with high job tenure (B = 0.13, *p* = 0.01). The model findings demonstrated that the model explained 39% of the variance (R^2^ = 0.39) and was statistically significant overall (F = 77.06, *p* < 0.001).

## 5. Discussion

This study examined the relationships between PJF and communication skills with patients and relatives expressing anger, as well as the moderating role of job tenure in these relationships. The findings of the present study indicated that PJF was positively associated with all subdimensions of communication skills with patients and relatives expressing anger. These findings align with prior research demonstrating the positive role of PJF in shaping employee attitudes and behaviors [15,30,31,32,33]. In particular, the relationship between PJF and communication skills with patients and relatives expressing anger was found to be stronger among employees with shorter job tenure across all subdimensions, except for CEM. In this regard, the findings suggest that the association between PJF and communication skills may vary depending on job tenure and highlight the important role of PJF in relation to employees’ communication skills. From this perspective, the present study extends the literature by demonstrating the role of PJF in the healthcare context, particularly in emotionally demanding interactions such as communication with patients and relatives expressing anger.

### 5.1. Implications for Theory

The theoretical implications of this study can be summarized as follows. First, the findings indicate that greater congruence between job requirements and employees’ knowledge, skills, and personal characteristics is associated with stronger communication competencies in interactions with angry patients. This finding suggests that PJF may serve as an important resource that enables more effective coping with emotionally demanding communication processes. These findings are consistent with COR theory, which posits that employees strive to protect and accumulate valuable resources [13]. In this context, employees who possess sufficient resources are more likely to develop functional and effective communication strategies by utilizing their existing resources, rather than perceiving emotionally demanding job requirements as threats [69]. For frontline administrative staff in healthcare services, PJF is an important personal resource and also functions as a protective psychological resource in situations involving high emotional demands, such as communication with angry patients.

Second, the study emphasizes the importance of job tenure in understanding the association between PJF and communication with angry patients and their relatives. The results revealed that the positive association between PJF and communication skills tends to diminish as job tenure increases. In other words, as job tenure increases, communication skills appear to rely more on experiential knowledge and established communication routines. At the same time, previous research has demonstrated that employees with higher levels of job tenure may improve their skills through learning from past interactions, developing coping strategies over time [70] and receiving guidance from more experienced colleagues [71]. Therefore, job tenure may function as a double-edged factor, providing experiential knowledge while also exposing employees to cumulative emotional demands. From a COR perspective, job tenure may be viewed as an accumulated resource that reshapes the relative importance of PJF in relation to communication skills. It may also moderate the strength of this relationship depending on contextual conditions.

At the same time, job tenure may not be considered solely as a positive resource; it may also entail potential negative consequences associated with prolonged exposure to emotionally demanding interactions. Individuals who work for extended periods in emotionally demanding environments may exhibit a tendency to rely on coping strategies based on their past experiences. Over time, such strategies may involve emotional suppression and avoidance when dealing with angry patients. With prolonged exposure, these patterns may increase the risk of burnout by draining psychological resources and reducing well-being [72,73,74]. Accordingly, this process may reduce emotional sensitivity and communication quality, helping to explain the overall weakening of the association observed at higher levels of job tenure.

Finally, the high correlations observed among the subdimensions of communication skills with angry patients may indicate some limitations in discriminant validity. Although the dimensions are theoretically distinct, this finding suggests a certain degree of empirical overlap among them. Nevertheless, the findings related to the CEM dimension are particularly noteworthy. While the other dimensions are more closely associated with managing interactions with angry patients and their relatives and regulating responses, the CEM dimension primarily reflects cognitive and skill-based processes involved in structuring verbal and nonverbal messages. As job tenure increases, these skills may become habitual through repeated experience, leading employees to rely more on learned communication behaviors rather than on PJF. This distinction may help explain why the moderating role becomes non-significant for the CEM dimension at higher levels of job tenure.

### 5.2. Implications for Practice

This study addresses frontline administrative healthcare staff’s communication skills with patients and relatives expressing anger within a multidimensional framework. It also links these skills to PJF, thereby extending an area that has received limited attention in the literature. Considering that frontline administrative healthcare staff in the Turkish context frequently encounter emotionally intense interactions in their communication processes with patients and their relatives [75], the findings of the present study offer important practice-oriented implications.

In this regard, it is important to inform prospective frontline administrative healthcare staff, prior to choosing the profession, that they are entering a field of work that requires patience, emotional resilience, and intensive interpersonal interaction. On the other hand, effective communication that can create a therapeutic effect between patients, their relatives, and healthcare personnel plays a decisive role in shaping patients’ emotional states, perceptions, and trust in healthcare services through healthcare workers’ verbal and nonverbal communication behaviors [44]. Therefore, encouraging frontline administrative healthcare staff to participate in in-service training programs, courses, and certification programs aimed at improving communication skills with patients and relatives expressing anger may help develop practical skills for managing emotionally demanding patient interactions. The findings of the present study suggest that employees with shorter job tenure may benefit from structured training, mentoring, and practical exercises (e.g., simulation- or case-based training) to better manage interactions with angry patients, whereas employees with longer job tenure may rely more on experience-based communication strategies (e.g., interpreting patients’ body language, responding calmly and professionally to patient complaints, and knowing when to appropriately conclude the interaction). Finally, promoting structured self-assessment practices that enable frontline administrative healthcare staff to regularly evaluate their professional roles and personal characteristics may contribute to strengthening PJF.

### 5.3. Implications for Management

Maintaining communication quality, particularly in interactions with angry patients and their relatives, requires managerial attention. In this context, the present study provides meaningful managerial implications for healthcare organizations by demonstrating that PJF constitutes an important antecedent of frontline administrative healthcare staff’s communication with patients and relatives expressing anger.

It is important that healthcare organizations do not confine in-service training policies for frontline administrative healthcare staff solely to the early stages of their careers. These trainings should instead be implemented periodically regardless of employees’ level of experience; for example, through communication-focused workshops or simulation-based communication training focusing on managing interactions with angry patients. In addition, recruitment and placement processes should be structured by considering the fit between the knowledge, skills, and competencies required by the position and the qualifications possessed by candidates [29]. Accordingly, healthcare organizations may be advised to assign frontline administrative healthcare staff not solely based on administrative requirements, but to units that are compatible with their personal characteristics and communication demands, particularly in departments characterized by frequent interactions with distressed patients, such as emergency units or patient registration desks. Finally, systematically collecting feedback from patients and their relatives within the scope of quality management may serve as a functional managerial tool. Sharing feedback obtained through patient and relative surveys or case-based evaluations with frontline administrative healthcare staff in a structured manner may help employees recognize their strengths as well as areas in need of improvement.

## 6. Study Limitations and Suggestions for Future Research

This research has certain limitations. First, the cross-sectional design restricts causal interpretations of the relationships among the variables. Second, the use of convenience and snowball sampling methods may introduce sampling bias and limit the generalizability of the findings. In addition, data were collected via online platforms such as LinkedIn and WhatsApp, which may have resulted in a sample skewed toward younger and more digitally active participants. Third, the data were collected using a single-source, self-report design. This may increase the risk of common method variance. Although procedural remedies were applied, future research should use multi-source data to reduce potential bias. Fourth, control variables such as age, gender and type of healthcare institution (public vs. private) were not included in the analysis. These factors may be associated with communication skills, and future research is encouraged to incorporate such variables to provide a more comprehensive understanding of these relationships. Fifth, job tenure was measured only in years of service. This may not capture the full complexity of experience. In addition, job tenure was analyzed in categories rather than as a continuous variable, which may limit the identification of a clear threshold effect. Future research should use more comprehensive measures. Another limitation is the high correlations among subdimensions. Although the dimensions theoretically reflect different behavioral skills, the Fornell–Larcker criterion was not fully satisfied, and some HTMT values exceeded the recommended threshold. The results may indicate limited discriminant validity. In this context, future research may examine the scale as a single construct. Additionally, as the use of a recently developed PJF scale may raise concerns regarding generalizability, future studies are encouraged to employ alternative measures of PJF. Finally, the sample was limited to frontline administrative healthcare staff in Turkey, and cross-cultural studies are needed to enhance the generalizability of the proposed model.

## 7. Conclusions

PJF was related to better communication with angry patients. Employees with higher PJF better understood patients’ emotions and gave more appropriate responses. Job tenure moderated this relationship. The relationship was stronger for employees with low and moderate tenure and weaker for those with high tenure. For CEM, the relationship was not significant at high levels of job tenure. Overall, PJF can be considered an important personal resource associated with communication skills, while job tenure shapes how these resources are used.

## Figures and Tables

**Table 1:** Descriptive Statistics for Continuous Variables.

Characteristics	Mean ± SE	Min–Max
Age	29 ± 0.67	20–50

Notes: Min = Minimum score observed; Max = Maximum score observed, SE = Standard error.

**Table 2:** Demographic Characteristics of the Participants.

Characteristics	Number (n)	Percentage (%)
Gender		
Female	199	54.8
Male	164	45.2
Marital status		
Married	76	20.9
Single	287	79.1
Educational status		
High school	7	1.9
Vocational school of health	314	86.5
Bachelor’s degree	36	9.9
Master’s degree/Doctorate	6	1.7
Job tenure		
1–5 years	186	51.2
6–10 years	138	38.0
More than 11 years	39	10.7
Type of Health Institution		
Public	123	33.9
Private	240	66.1
Department		
Outpatient Clinic	189	52.1
Inpatient Clinic	81	22.3
Emergency Department	27	7.4
Registration Desk	66	18.2

**Table 3:** Reliability Coefficients of the Scales.

Constructs	Loadings	Cronbach’s α	CR	AVE
PJF	0.82–0.93	0.909	0.94	0.79
DPA	0.75–0.94	0.932	0.95	0.79
CEM	0.78–0.88	0.896	0.92	0.71
AAA	0.73–0.83	0.799	0.87	0.62
AM	0.70–0.90	0.895	0.93	0.72

Notes: Cronbach’s α = Cronbach alfa; CR = Composite reliability; AVE = Average variance extracted.

**Table 4:** Confirmatory Factor Analysis Results of the Person–Job Fit Scale and the Communication Skills with Angry Patients and Their Relatives Scale.

Goodness of Fit Indices	Perfect Fit Criteria	Acceptable Fit Criteria	PJF	CSAP	Evaluation
χ^2^/df	0 ≤ χ^2^/df ≤ 3	3 ≤ χ^2^/df ≤ 5	3.33	3.02	Acceptable
CFI	0.95 ≤ CFI	0.85 ≤ CFI	0.99	0.94	Acceptable
GFI	0.90 ≤ GFI	0.80 ≤ GFI	0.99	0.86	Acceptable
AGFI	0.90 ≤ AGFI	0.80 ≤ AGFI	0.95	0.82	Acceptable
RMSEA	0 ≤ RMSEA < 0.05	0.06 ≤ RMSEA ≤ 0.10	0.08	0.07	Acceptable

Notes: χ^2^ = Pearson Chi-Square; df = Degrees of freedom; CFI = Comparative fit index; GFI = Goodness of fit index; AGFI = Adjusted goodness of fit index; RMSEA = Root-mean-square error of approximation; Person–Job Fit = PJF; CSAP = Communication Skills with Angry Patients.

**Table 5:** Descriptive Statistics.

Constructs	n	Min	Max	$\bar{x}$	SD	Skewness	Kurtosis
PJF	363	1.00	5.00	3.40	0.96	−0.48	−0.27
Communication Skills with Angry Patients and Their Relatives	363	1.00	5.00	3.87	0.77	−0.70	−0.02
DPA	363	1.00	5.00	3.89	0.87	−0.65	0.06
CEM	363	1.00	5.00	3.88	0.80	−0.58	−0.14
AAA	363	1.00	5.00	3.86	0.78	−0.69	0.73
AM	363	1.00	5.00	3.86	0.86	−0.67	0.06

Notes: n = Number of participants; Min = Minimum score observed; Max = Maximum score observed, $\bar{x}$ = Mean, SD = Standard deviation.

**Table 6:** Correlation Matrix.

Scales and Sub-Dimensions	1	2	3	4	5
1. PJF	1				
2. DPA	0.46 **	1			
3. CEM	0.38 **	0.88 **	1		
4. AAA	0.37 **	0.80 **	0.80 **	1	
5. AM	0.44 **	0.85 **	0.80 **	0.77 **	1

Notes: n = 363; ** *p* < 0.01.

**Table 7:** Regression Analysis Results for Variables.

Variables	Unstandardized Coefficients		Standardized Coefficients			Confidence Interval	
	B	SE	β	t	*p*	LLCI	ULCI
Model 1 (DPA)							
Constant	2.451	0.150		16.336	<0.001	2.156	2.746
PJF	0.423	0.042	0.464	9.963	<0.001	0.340	0.507
F = 99.262, R = 0.464, R^2^ = 0.216, adjusted R^2^ = 0.213, *p* < 0.001							
Model 2 (CEM)							
Constant	2.815	0.143		19.656	<0.001	2.533	3.097
PJF	0.314	0.041	0.378	7.752	<0.001	0.235	0.394
F = 60.095, R = 0.378, R^2^ = 0.143, adjusted R^2^ = 0.140, *p* < 0.001							
Model 3 (AAA)							
Constant	2.850	0.141		20.227	<0.001	2.572	3.127
PJF	0.298	0.040	0.366	7.464	<0.001	0.219	0.376
F = 55.706, R = 0.366, R^2^ = 0.134, adjusted R^2^ = 0.131, *p* < 0.001							
Model 4 (AM)							
Constant	2.532	0.150		16.832	<0.001	2.236	2.827
PJF	0.392	0.043	0.436	9.211	<0.001	0.309	0.476
F = 84.845, R = 0.436, R^2^ = 0.190, adjusted R^2^ = 0.188, *p* < 0.001							

Notes: B = Unstandardized regression coefficient; SE = standard error; β = standardized regression coefficient; t = t-statistic; *p* = significance level; LLCI = 95% lower-limit confidence interval; ULCI = 95% upper-limit confidence interval.

**Table 8:** Regression Analysis Results Showing the Moderating Effect.

Variables		B	SE	t	*p*	LLCI	ULCI
Model 1: DPA (Y)							
Constant		0.09	0.34	0.25	0.80	−0.59	0.77
PJF (X)		0.90	0.10	8.88	<0.001	0.70	1.10
Job tenure (W)		1.61	0.20	8.25	<0.001	1.23	2.00
X × W		−0.33	0.06	−5.97	<0.001	−0.44	−0.22
	Low	0.57	0.05	10.50	<0.001	0.46	0.68
Job tenure	Moderate	0.38	0.04	9.76	<0.001	0.30	0.45
	High	0.15	0.05	3.13	<0.001	0.06	0.25
Model summary		F = 83.25, R = 0.64, R^2^ = 0.41, df1 = 3, df2 = 359, *p* < 0.001					
Model 2: CEM (Y)							
Constant		0.67	0.33	2.02	0.04	0.02	1.32
PJF (X)		0.74	0.10	7.58	<0.001	0.55	0.94
Job tenure (W)		1.47	0.19	7.76	<0.001	1.10	1.84
X × W		−0.30	0.05	−5.57	<0.001	−0.40	−0.19
	Low	0.45	0.05	8.49	<0.001	0.34	0.55
Job tenure	Moderate	0.27	0.04	7.25	<0.001	0.20	0.34
	High	0.07	0.05	1.46	0.14	−0.02	0.16
Model summary		F = 61.41, R = 0.58, R^2^ = 0.34, df1 = 3, df2 = 359, *p* < 0.001					
Model 3: AAA (Y)							
Constant		1.29	0.35	3.74	<0.001	0.61	1.97
PJF (X)		0.60	0.10	5.86	<0.001	0.40	0.80
Job tenure (W)		1.08	0.20	5.49	<0.001	0.69	1.47
X × W		−0.21	0.06	−3.81	<0.001	−0.32	−0.10
	Low	0.39	0.06	7.06	<0.001	0.28	0.49
Job tenure	Moderate	0.26	0.04	6.72	<0.001	0.18	0.34
	High	0.12	0.05	2.38	0.02	0.02	0.21
Model summary		F = 40.84, R = 0.50, R^2^ = 0.25, df1 = 3, df2 = 359, *p* < 0.001					
Model 4: AM (Y)							
Constant		0.22	0.34	0.63	0.53	−0.46	0.89
PJF (X)		0.85	0.10	8.37	<0.001	0.65	1.05
Job tenure (W)		1.59	0.20	8.10	<0.001	1.20	1.98
X × W		−0.32	0.06	−5.77	<0.001	−0.43	−0.21
	Low	0.53	0.06	9.76	<0.001	0.43	0.64
Job tenure	Moderate	0.34	0.04	8.87	<0.001	0.27	0.42
	High	0.13	0.05	2.58	0.01	0.03	0.22
Model summary		F = 77.06, R = 0.63, R^2^ = 0.39, df1 = 3, df2 = 359, *p* < 0.001					

Notes: Bootstrap analyses were conducted using 5000 resamples; B = unstandardized regression coefficient; SE = standard error; t = t-statistic; *p* = significance level; LLCI = lower limit of the 95% confidence interval; ULCI = upper limit of the 95% confidence interval.

## Data Availability

Due to participant privacy obligations and applicable ethical restrictions, the datasets generated and analyzed during the current study are not publicly accessible. Access may be granted upon reasonable request to the corresponding author, contingent on a justified purpose and compliance with relevant ethical and legal requirements.

## Abbreviations

The following abbreviations are used in this paper:

PJF	Person–job fit
DPA	Displaying a positive approach
CEM	Creating effective messages
AAA	Anger anticipation and awareness
AM	Anger management
COR	Conservation of resources
